# An approach to rapid processing of camera trap images with minimal human input

**DOI:** 10.1002/ece3.7970

**Published:** 2021-08-02

**Authors:** Matthew T. Duggan, Melissa F. Groleau, Ethan P. Shealy, Lillian S. Self, Taylor E. Utter, Matthew M. Waller, Bryan C. Hall, Chris G. Stone, Layne L. Anderson, Timothy A. Mousseau

**Affiliations:** ^1^ Department of Biological Sciences University of South Carolina (UofSC) Columbia South Carolina USA; ^2^ South Carolina Army National Guard Environmental Office Eastover South Carolina USA

**Keywords:** camera trap, deep learning, neural network, transfer learning, wildlife ecology

## Abstract

1. Camera traps have become an extensively utilized tool in ecological research, but the manual processing of images created by a network of camera traps rapidly becomes an overwhelming task, even for small camera trap studies.
2. We used transfer learning to create convolutional neural network (CNN) models for identification and classification. By utilizing a small dataset with an average of 275 labeled images per species class, the model was able to distinguish between species and remove false triggers.
3. We trained the model to detect 17 object classes with individual species identification, reaching an accuracy up to 92% and an average F1 score of 85%. Previous studies have suggested the need for thousands of images of each object class to reach results comparable to those achieved by human observers; however, we show that such accuracy can be achieved with fewer images.
4. With transfer learning and an ongoing camera trap study, a deep learning model can be successfully created by a small camera trap study. A generalizable model produced from an unbalanced class set can be utilized to extract trap events that can later be confirmed by human processors.

## Graphical abstract

We have streamlined an approach for adopting artificial intelligence methods for the rapid identification of animals from camera trap images. We use this rapid approach to document the abundance and species richness of animals living on two National Guard military sites in South Carolina.

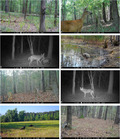

## 1 INTRODUCTION

Observational studies of wildlife occupancy and abundance are more important than ever as human disturbance has decreased wildlife population sizes by up to 60% globally in the last four decades (WWF, 2018). These staggering declines have prompted the establishment of ecological monitoring through a variety of means including camera traps, mark–recapture methods, point counts, and line transects. Camera traps have become an especially useful survey methodology for the rapid assessment of wildlife because they require fewer field hours than other common field methods, may be reviewed by other researchers, and minimize disturbance to the environment (McCallum, 2013; Silveira et al., 2003; Steenweg et al., 2017). While camera traps are a useful tool for some ecological studies, processing massive quantities of images created by camera trap networks is a major limiting factor for humans. Until methods are developed for the common camera trap study that does not have a sufficient number of images to train a new model, human processing limitations will persist in future studies and only worsen as camera trap projects become more complex.

Previous camera trap studies have noted factors which increase the number of false camera triggers, resulting in large accumulations of images. Wind, loose shrubbery, camera settings, and animal behavior specific to each camera site add noise to the dataset (Newey et al., 2015). The time involved in manually processing these false triggers, which often represent a majority of captured images, can delay analysis to the point where conclusions are no longer relevant. Often, important metrics are left underexplored or unaccounted for all together because a large expenditure of resources is often required to process images manually (Willi et al., 2019).

Increase in the use of camera traps for ecological studies has led to a push for standardized methods to improve the workflow of image analysis (Glover‐Kapfer et al., 2019). One promising avenue for processing camera trap images is the utilization of artificial intelligence (AI) technology. Artificial neural networks (ANNs) are AI algorithms which are composed of nodes or “neurons” stratified into layers. In the case of image classification, “training” occurs when a set of images is fed into the algorithm along with their known classifications, and the model assigns weights to features at multiple levels of abstraction which it identifies to be important in recognizing the object(s) specified in the image. In the case of image recognition and classification, the base‐level features extracted from the image are red, green, and blue (RGB) values for each pixel. The RGB values are passed to deeper layers of the neural net which use the distribution of these values to identify more complex components of the image, such as contours and shapes. Once a model is sufficiently trained, it can utilize the weights extracted from the training data to make predictions about the contents of novel “test” images.

Convolutional neural networks (CNNs) build upon the traditional ANN structure by “convoluting” images prior to analysis. Convolution consists of a matrix operation which effectively reduces the precise resolution of the images, leading to less overall connections between nodes and thus a more generalizable set of image features, without significantly sacrificing performance (Krizhevsky et al., 2017). The structure of CNNs makes them an ideal candidate to enhance the generalizability and inhibit overfitting to a specific image set. Overfitting is a phenomenon that occurs when a model cannot be generalized to the test set during training; therefore, it is not generalizable to the remainder of the images in the study and certainly not images of the same environment in different studies.

AI trained with convolutional neural networks (CNNs) has been employed and tested on several large datasets previously processed by citizen scientists. Swanson et al. (2015) trained and created a CNN for the Snapshot Serengeti dataset which consists of 3.2 million images collected over 99,241 camera trap days. The output of the neural network reached an accuracy of greater than 93.8% when compared to the records of citizen scientists. While several large‐scale studies (e.g., Norouzzadeh et al., 2018) have achieved similar accuracy on such large datasets, the training of these neural networks requires large numbers of images and substantial computer time to train the model. Such investments are often not feasible for smaller camera trap studies under the current assumption that many thousands of images are needed to successfully train a model.

Only the largest camera trap studies have attempted to create their own neural networks, as it has been suggested that small clusters of images (~1,000–5,000 images per species class) are not sufficient for deep learning (e.g., Norouzzadeh et al., 2018). In order for a small camera trap study to utilize these models, they would need to augment their own large image set of a particular species or distractive environmental backgrounds that lead to false identifications (e.g., vehicles, flora, and livestock). The additional input to use these methods, although worth the effort to have a diverse and generalizable model already trained, limits the feasibility of this approach for small studies. Here, we provide an alternative approach that requires significantly fewer images by utilizing transfer learning and bounded‐box labeling. CNNs learn the features belonging to each species class, allowing it to differentiate between objects and the background of images while also classifying objects. This alternative method would address the concern of image sets not being similar enough to another study's range of objects and backgrounds to be useful, even in the same geographical location.

Transfer learning, or transfer training, is a machine‐learning technique that uses feature maps already trained on previous, similar datasets. This tactic requires less training with new image sets because it is already capable of identifying lower‐level patterns common between the sets of images. In other words, the important features extracted from the labeled domains of the past training data give a head‐start in training on the new images, therefore requiring fewer images to train effectively (Shao et al., 2015). This type of training is used in other camera trap studies, but to our knowledge has not been previously applied to small studies such as our own. However, similar studies completed in the medical field have shown that given scarce data, transfer learning is more accurate than other state‐of‐the‐art methods (Deepak & Ameer, 2019; Swati et al., 2019) and has been effective in false‐positive reduction (Shi et al., 2019).

We suggest that the use of transfer learning on neural networks is often overlooked for small‐scale camera trap studies (Schneider et al., 2020). Adapting a neural network to a dataset by adjusting the output of the final layers of the network through transfer learning and then reinforcement learning on a desired image set can be extremely useful, especially when data are scarce. We predict that a premade neural network, utilizing the process of transfer learning, could achieve similar identification accuracy as neural networks trained with thousands of images while not requiring such a large memory footprint. Using a transfer‐trained neural network that may only need a few thousand images (depending on the complexity of the object) allows camera trap surveys to be affordable, data efficient, and accessible to a broad range of projects.

Neural networks are used for various types of image processing and many are freely available through open‐source software (e.g., Google, PyTorch, Keras). A premade neural network can be selected from an archive based on the types of images the network was built on; for instance, a neural network trained on animals/pets would be ideal for a camera trap project interested in identifying medium‐ to large‐sized mammals. To mimic a small‐scale camera trap study, we trained a premade, freely available neural network on the Faster‐RCNN architecture using less than 6,000 images from our larger dataset and achieved similar confidence in object identification as the previously mentioned large‐scale studies. Here, we show that a small number of diversified images can be just as successful at eliminating false positives and identifying species as a model developed using many thousands of images.

## 2 METHODS

### 2.1 Camera trap study

**FIGURE 1:**
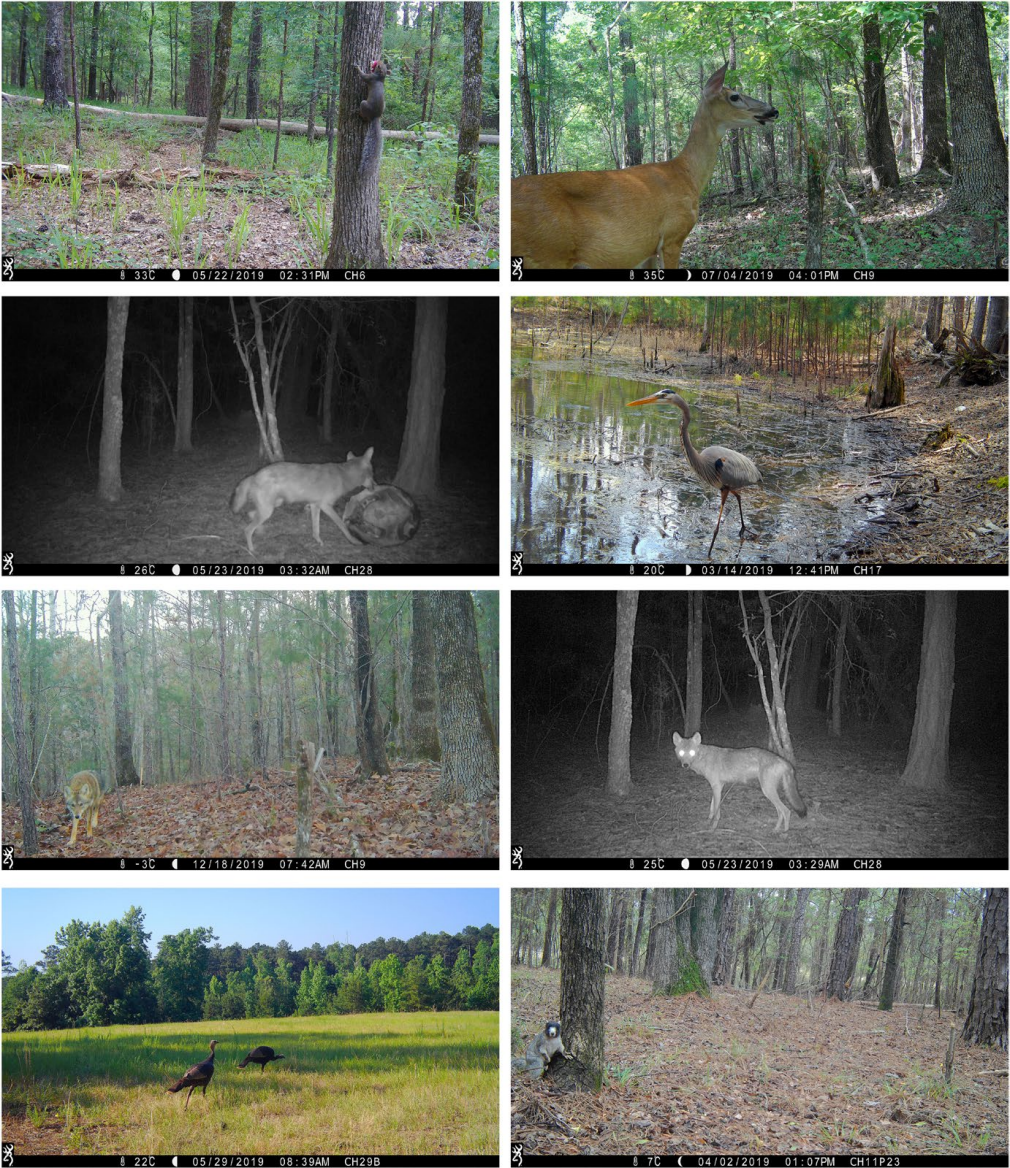
Sample photographs from camera traps. Starting from top left and going clockwise, species are as follows: Carolina gray squirrel (*Sciurus carolinensis)*, white‐tailed deer (*Odocoileus virginianus),* great blue heron (*Ardea herodias*), coyote (*Canis latrans*), fox squirrel (*Sciurus niger*), wild turkey (*Meleagris gallopavo*), and coyote (*Canis latrans*)

The subset of images used to train the model was pulled from a camera trap study consisting of 170 cameras, which were deployed for up to three years across two regions of South Carolina (see Appendix S1 for camera trap study details). Some examples of images obtained are shown in Figure 1. We acquired images for the train and test datasets from 50 camera locations from each region within two separate one‐month time frames. The complete test and train datasets consisted of 5,277 images of 17 classes, including images from both winter and summer months to account for seasonal background variation (Table 1). True‐negative images were not included because they would not assist in teaching the model about any of the species classes. A commonly used 90/10 split (e.g., Fink et al., 2019) was utilized to create the training and testing datasets from the selected images; 90% of images were used for training and 10% were used for testing.

**TABLE 1:** Distribution of image subset for train and test datasets by class

Class	Train		Test	
	Images	Objects	Images	Objects
Armadillo	186	186	21	21
Bobcat	18	18	4	4
Coyote	162	171	18	18
Crow	39	59	11	13
Deer	1,109	1,379	136	159
Dog	86	114	18	21
Fox Squirrel	79	79	17	18
Gray Fox	88	88	11	11
Gray Squirrel	318	327	32	34
Heron	52	52	3	3
Human	822	1,948	89	194
Opossum	18	18	3	3
Rabbit	269	278	17	17
Raccoon	200	208	26	26
Skunk	17	17	2	2
Turkey	430	879	43	80
Vehicle	780	2,962	84	271
Total	4,673	8,783	535	895

### 2.2 Image selection

**FIGURE 2:**
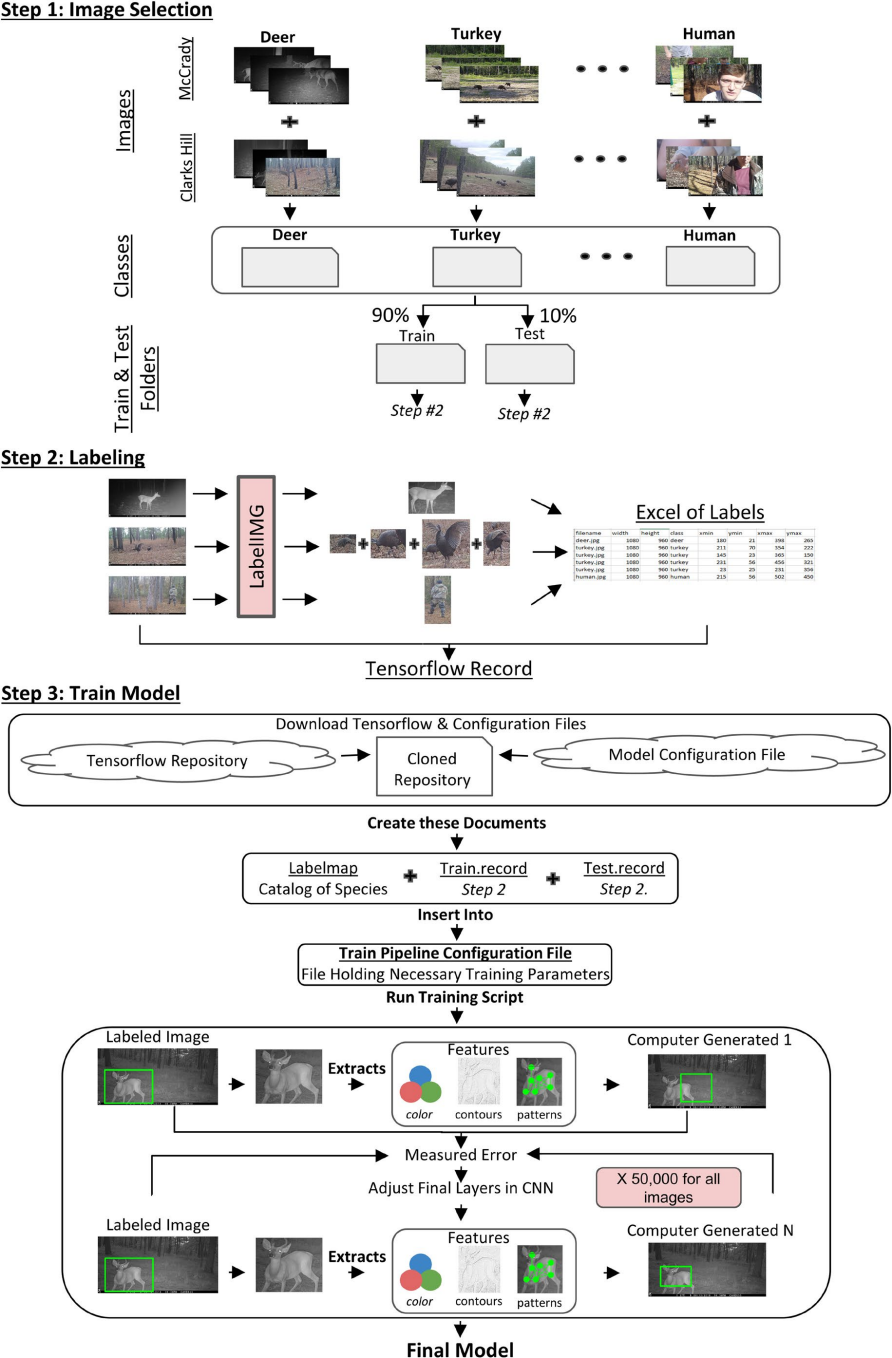
Diagram of image collection and training process. The visual representation demonstrates the main ideas of selecting and organizing up to 500 images for each class, employing transfer learning, and producing the final identification model that is set to classify animals within the camera trap study

The basic process of designing an identification and classification model (Figure 2) included selecting and labeling a subset of images from our camera trap image repository (see Appendix S1 for details) for transfer learning, in order to adapt a premade neural network to our image set. The subset of images used to train the model was pulled from a camera trap study consisting of 170 camera stations which had been deployed for up to three years in two regions of South Carolina (see Appendix S1 for camera trap study details). To begin, a subset of images was created by selecting up to 500 images of each species from the South Carolina Army National Guard (SCARNG) training centers in a variety of positions within the field of view (Figure 2, Step 1). In cases where classes (species being classified) reached 500 images, only images that contributed a unique perspective of the animal were added to the training dataset, in order to supply the model with a better generalization of the animal and prevent class imbalance. Despite adding more than 500 images to some classes, the model did not seem to favor one class over the other.

### 2.3 Feature extraction

To get the most out of the small image set, every object within each image was labeled for supervised training (Figure 2, Step 2) (Dai et al., 2015). The use of supervised training increased the accuracy of detection and classification by providing a well‐defined region of interest for each object in the image through human‐generated bounding boxes (Appendix S2). LabelImg (Tzutalin, 2015), a graphical image annotation tool, was used to establish ground truths (locations of all objects in an image) and create the records needed for our supervised training process. This software allows a user to define a box containing the object and automatically generates a CSV file with the coordinates of the bounding box as well as the class defined by the user.

### 2.4 Classification training

A transfer learning process to adapt a premade neural network (Figure 2, Step 3) was utilized to create an identification and classification model. We transformed the CSV file generated by the feature extraction process into a compatible tensor dataset for the training process through the appropriate methodologies laid out in the Tensorflow (Abadi et al., 2015) package description. Tensorflow is an open‐source, experimental Python library from Google for identification and classification models. The Tensorflow transfer learning process required a clone of the Tensorflow repository, in combination with a customized model configuration file defining parameters (Table 2).

**TABLE 2:** Details about model training and hardware used

CPU	Windows 10 Intel i9‐9
RAM	64 GB
GPU	Nvidia 2070 super 8 GB
Batch size (images per training round)	4
Epoch steps (complete cycle through training data)	50,000
Train configuration	Faster R‐CNN Inception v2
Training evaluation	Every 1,000 steps
Evaluation configuration	Open Images V2 Detection Metric

### 2.5 Training evaluation

The degree of learning that was completed after each step was analyzed using intersection over union (IOU) as training occurred (Krasin et al., 2017). A greater IOU equates to a higher overlap of generated predictions versus human‐labeled regions, thus indicating a better model (see Appendix S3). Observing an asymptote in IOU allowed for the determination of a minimum number of steps needed to train the model for each class and to assess which factors influenced the training process (e.g., feature qualities, amount of training images). Because the minimum step number was not associated with image quantity in determining step requirements, we relied on quality assessments, such as animal size and animal behavior.

**FIGURE 3:**
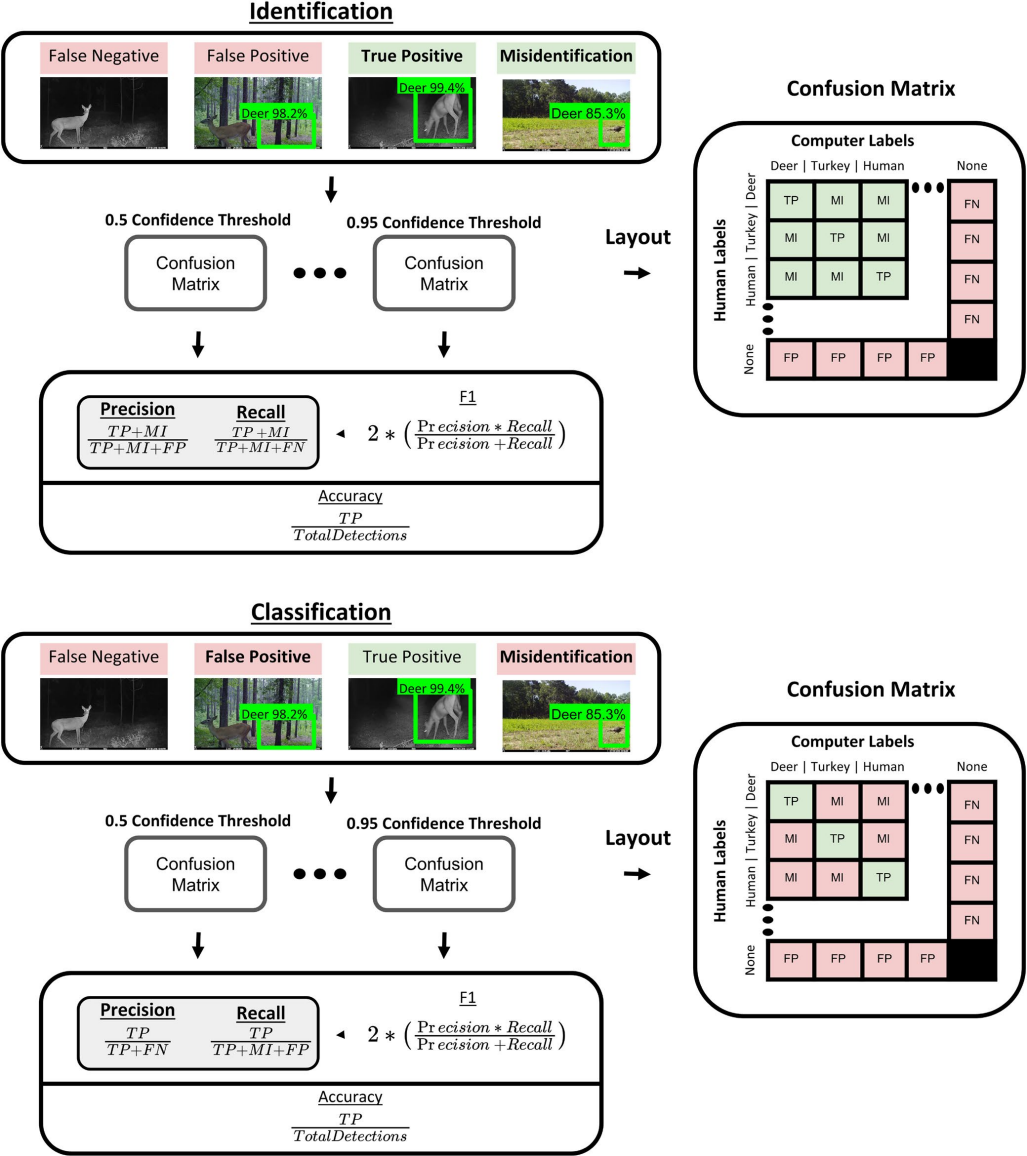
Diagram illustrating calculation of each metric used in training (train and test) data: precision, recall, accuracy, and F1 (range of 0–1). For identification purposes, misidentifications are counted as correct (green in confusion matrix) because the animal was detected, whereas, for classification purposes, misidentifications are counted as incorrect (red in confusion matrix) because the object was not classified correctly. True positives (TP), false positives (FP), and false negatives (FN) are represented in the confusion matrix with true negatives (TN) not present in training data. Adjusting confidence thresholds (range of 0.5–0.95) optimizes the model for specific applications

Following training, final discrepancies between the model output and the labeled ground truths were summarized into confusion matrices (generated by scikit‐learn, Table 3) including false positives (FP), false negatives (FN), true positives (TP), true negatives (TN), and misidentifications (MI) (Table 4). Several metrics were calculated to evaluate aspects of model performance (Figure 3). Relying on accuracy alone may result in an exaggerated confidence in the model's performance, so to avoid this bias, the model's precision, recall, and F‐1 score were also calculated. Precision is a measure of FPs while recall is a measure of FNs, with F‐1 being a summary of the two metrics (Figure 3). Due to the large proportion of TNs associated with camera trap studies, F‐1 score does not include TNs in order to focus on measuring the detection of TPs.

**TABLE 3:** Confusion matrix of predicted versus ground truth values example for training of 17 object classes at 0.9 confidence threshold (CT). Color gradient from red to green indicates the number of detections. Yellow is intermediate

	Predicted values																	
	Armadillo	Bobcat	Coyote	Crow	Deer	Dog	Fox Squirrel	Gray Fox	Gray Squirrel	Heron	Human	Opossum	Rabbit	Raccoon	Skunk	Turkey	Vehicle	FN
*Ground truth values*																		
Armadillo	18																	3
Bobcat			1															3
Coyote			8		3				1					1				5
Crow				6														6
Deer			1		136	1					2		1					18
Dog					2	11												8
Fox Squirrel							6		3									9
Gray Fox								10										1
Gray Squirrel					1				11									22
Heron										3								
Human											158							36
Opossum														1				2
Rabbit					1								11					5
Raccoon														19				6
Skunk															2			
Turkey											1					71		8
Vehicle						1											231	39
FP	1				8	1		1	4	1	47					1	34	

**TABLE 4:** True‐positive (TP), false‐positive (FP), false‐negative (FN), and true‐negative (TN) values for completed training and validation at 0.9 confidence threshold (CT) for the 17 object classes

Species	Training			Validation		
	TP	FP	FN	TP	FN	FP
Armadillo	18	1	3	0	0	0
Bobcat	0	0	3	0	0	4
Coyote	18	0	5	7	11	15
Crow	6	0	6	0	0	0
Deer	136	8	18	1,016	127	235
Dog	11	1	8	0	0	7
Fox Squirrel	6	0	9	1	2	36
Gray Fox	10	1	1	0	0	8
Gray Squirrel	11	4	22	0	3	59
Heron	3	1	0	0	0	1
Human	158	47	36	27	2	88
Opossum	0	0	2	0	0	0
Rabbit	11	0	5	0	0	2
Raccoon	19	0	6	1	6	1
Skunk	2	0	0	0	0	0
Turkey	71	1	8	0	8	20
Vehicle	231	34	39	114	33	72
Total TN	0			9,499		

In addition, the metrics were further separated into evaluations for identification and classification purposes. Identification (ID) models would focus only on finding objects and therefore deem misidentifications as correct because the object was found. Classification (CL) models would not deem misidentifications as correct. Finally, accuracy, precision, recall, and F‐1 were calculated at a variety of confidence thresholds (CT), a parameter constraining the lower limit of confidence necessary for a classification proposal, to determine the threshold that resulted in the highest value of the metric we wished to optimize.

### 2.6 Validation

To confirm results acquired from testing the model, it was essential to evaluate a validation set of images. This validation set was formed by randomly selecting five cameras from a 12‐week period separate from the training dataset, but within the same larger dataset. The validation subset consisted of 10,983 images, including true negatives. The set ran using the optimal CT for F‐1 score determined by the test data. These images were also labeled using LabelImg to automate the calculation of evaluation metrics. The validation set scores and test scores should be compared to determine whether the model is overfitted, meaning the test set is not representative of the validation set. Possible reasons for such a mismatch may be that the background environment has changed dramatically or species not included in the test set have appeared.

## 3 RESULTS

### 3.1 Evaluation of training

**FIGURE 4:**
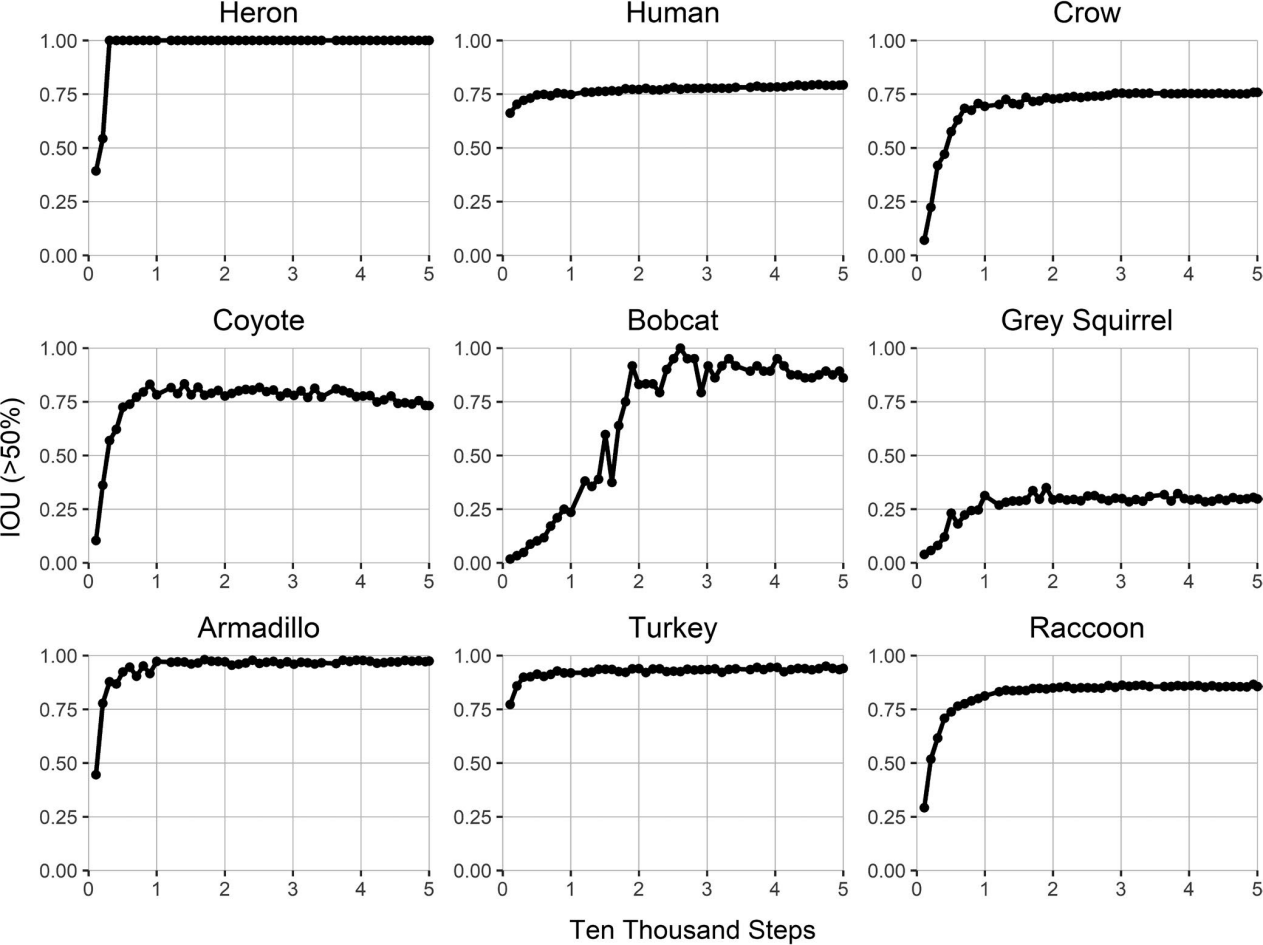
Average precision at 50% intersection over union found every 10,000 steps for select classes. Graphs for all species can be found in Appendix S3

**FIGURE 5:**
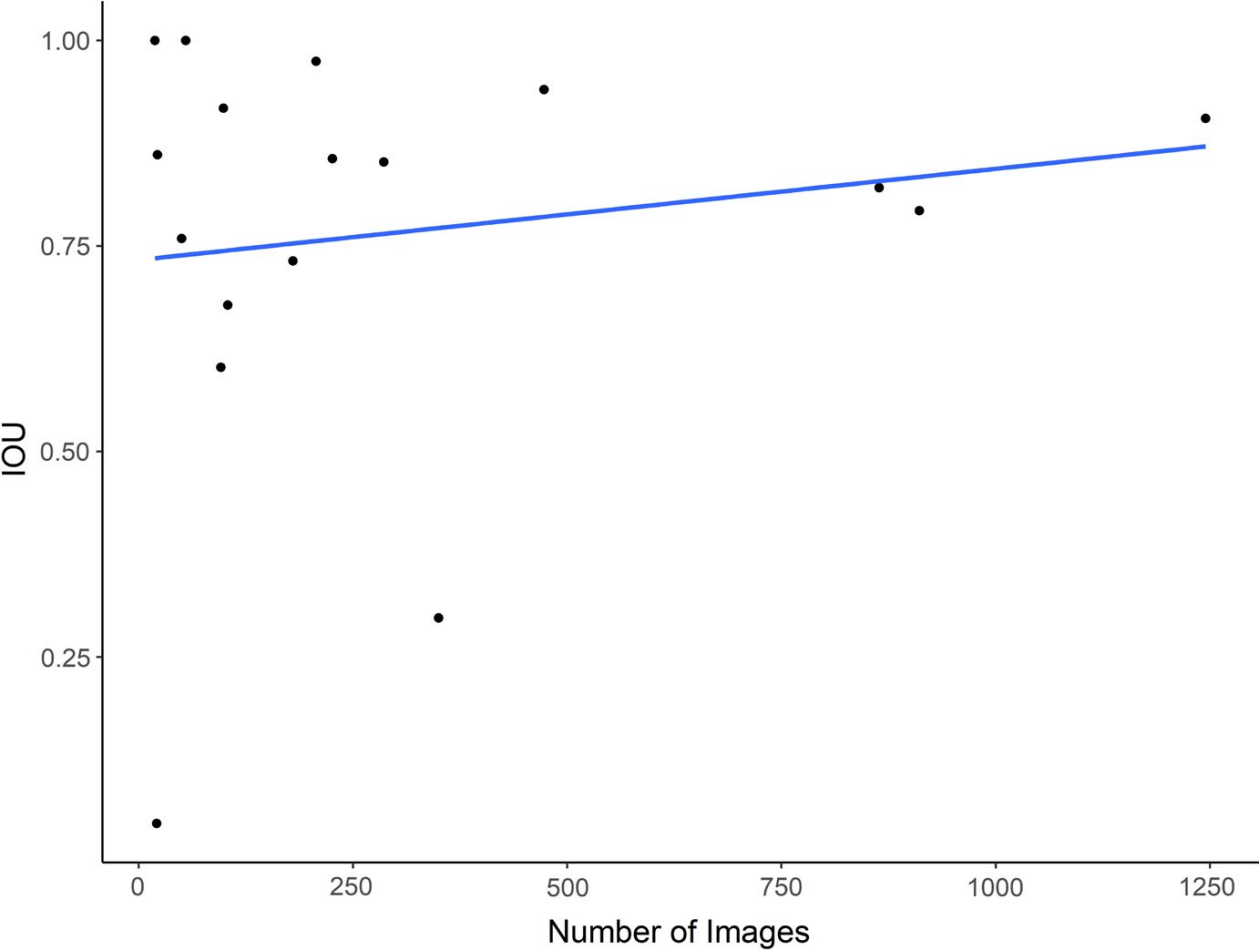
Evaluation of image size versus final intersection over union values for each of the 17 object classes ordered by number of images (blue) used for training. Linear regression model of number of images versus IOU (*y* = 0.0001*x* + 0.7330; *R* ^2^ = 0.0254; *p*‐value = .5409). Spearman correlation test supports no correlation between IOU and number of images (*ρ* = −0.017, *p* value = .948)

The performance of our model did not depend on the number of images used to train each species class (Figure 5). In fact, precision during the training process varied greatly among species classes and was not a function of the number of images input into the model (Figure 4). The class with the highest precision during training was armadillo (98%) with 186 images while gray squirrel had the lowest precision during training (30%), despite being trained on 318 images. The raccoon, turkey, and deer classes all resulted in comparably high precision values while being trained using 88, 430, and 1,109 images, respectively (Figure 4). Five classes were trained using less than 60 images between the test and train dataset (Table 2, see Appendix S3 for all IOU graphs). Result metrics for these classes also varied as a function of species traits rather than number of images used to train the class (*R* ^2^ = 0.0251, Figure 5).

### 3.2 Model performance

**FIGURE 6:**
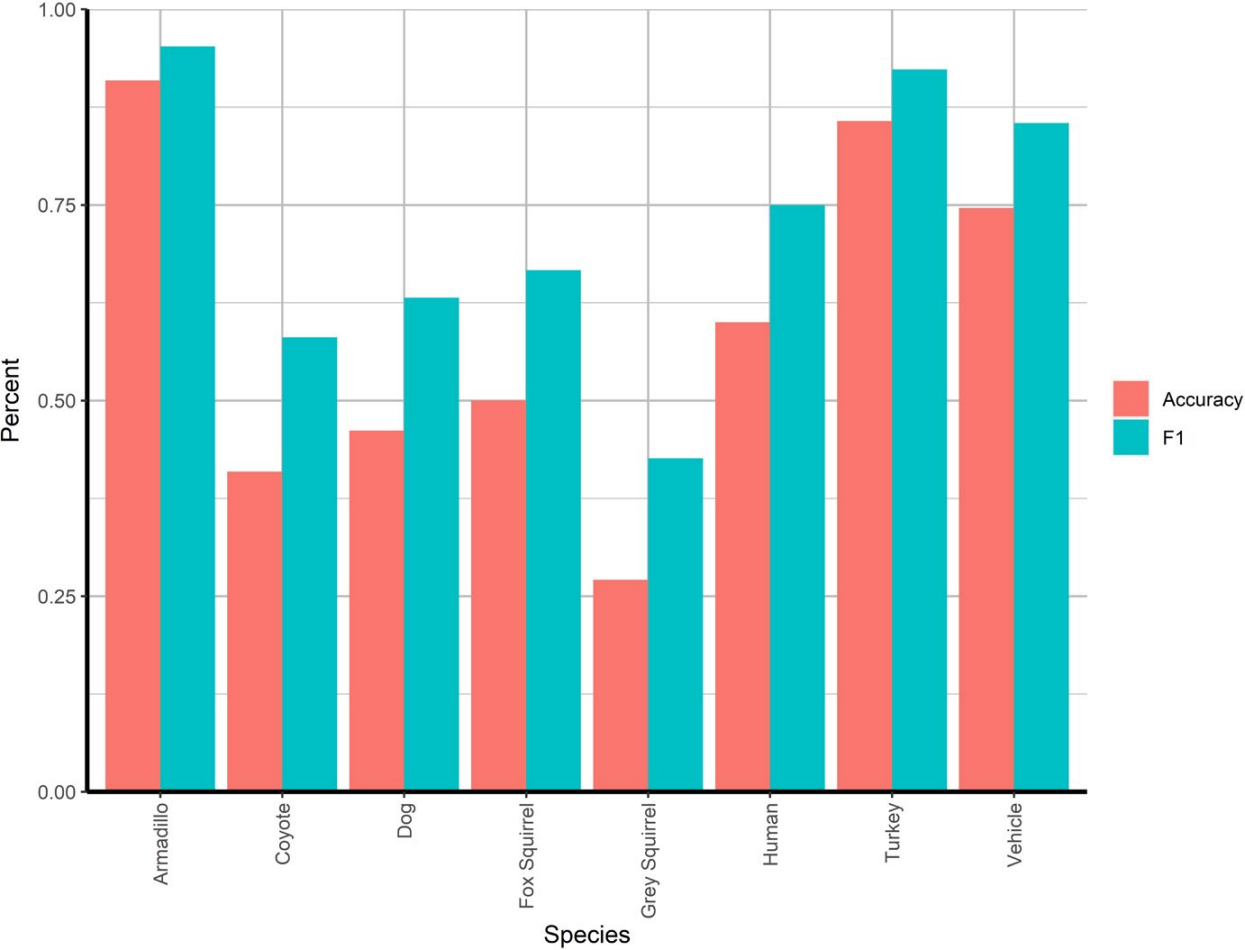
Comparison of select classes at 0.95 confidence threshold (CT) from test output. F‐1 values (white) are consistently higher than the accuracy (black)

To judge the performance of the model, we evaluated accuracy, precision, recall, and F‐1 at several CTs using the corresponding TP, FP, TN, and FN values (Table 4); these values were calculated from the respective confusion matrices (e.g., Table 3). Metrics followed the same trends for both ID and CL purposes with CL values running slightly below ID values (Table 5). The test set produced recall values that were inversely related to the CTs, while the precision values were directly related; precision was highest at 0.95 CT (ID: 90%, CL: 88%) and recall was highest at 0.50 CT (ID: 96%, CL: 89%). Accuracy for identification was highest at the 0.50 CT, and accuracy for classification was highest at the 0.90 CT (ID: 75%, CL: 71%). F‐1 score was highest at the 0.70 CT for ID (86%) and 0.90 CT for CL (83%). The difference between accuracy and F‐1 values demonstrates the effect of TNs (Figure 6). Accuracy and F‐1 were highest at 0.90 CT for the test data; therefore, we decided to use 0.90 CT for the validation set. The validation test resulted in a 93% accuracy, 68% precision, 86% recall, and 76% F‐1 score (Table 5).

**TABLE 5:** Summary of averages at each confidence threshold (CT)

Confidence threshold	Test—Identification				Test—Classification			
	Accuracy	Precision	Recall	F−1	Accuracy	Precision	Recall	F−1
0.50	75	75	96	84	64	70	89	78
0.60	71	79	94	85	67	74	88	80
0.70	72	81	91	86	68	77	86	81
0.80	73	84	88	86	69	80	84	82
0.90	73	88	83	85	71	85	80	83
0.95	71	90	78	84	69	88	77	82

Validation—Classification	Accuracy	Precision	Recall	F−1
0.90 confidence threshold	92	68	86	76

## 4 DISCUSSION

### 4.1 CNN accessibility

This study demonstrates that CNN‐based identification and classification models are more accessible than previously thought. Processing of camera trap images has been limited by human observers, expense, processing time, and ignorance of computer science techniques for applications in ecological studies. Employing labeling services (e.g., Google Cloud) can be unreliable for processing large datasets and to have images labeled and processed currently costs approximately $0.05 per image (Google Cloud), which may not be practical when tens of thousands of images are involved.

An increasingly accurate and efficient method of image processing is transfer learning (e.g., Deepak & Ameer, 2019; Shi et al., 2019; Swati et al., 2019), which is an especially desirable technique for studies with limited data (Shin et al., 2016). Despite improvements in this training architecture, the use of these methods in ecology has been limited. Transfer learning saves time and reduces data requirements, allowing for smaller studies to spend less time processing while still calibrating the architecture with specific images and training the model on a percentage of their complete dataset. Additionally, transfer learning helps prevent overfitting of the model, which can be an issue when using a smaller number of images (Deepak & Ameer, 2019; Han et al., 2018; Schneider et al., 2020).

A smaller image set allows the model to be more flexible, making it more applicable for ecologists than other advanced machine learning techniques (Xie et al., 2015). Feature extraction with transfer learning provides camera trap projects an alternative option to starting a CNN architecture from scratch, instead opting to use a pre‐trained CNN product (e.g., Microsoft MegaDetector) or unsupervised learning techniques (e.g., cluster analysis).

By using open‐source programs and calibrating premade neural nets, models can be built to simply remove images without animals or to fully automate the classification of species. This study, along with similar studies (e.g., Tab ak et al., 2018), provides evidence that a reliable identification and classification model can be created with open‐source tools (e.g., Tensorflow) by using transfer learning and premade neural networks (see Appendix S4). Further, we completed this process using a very limited set of images and achieved encouraging results. This technology could be especially desirable for researchers wishing to eliminate false positives as well as to quickly sort and label species classes.

### 4.2 Calibration analysis

Currently, accuracy is the standard metric to evaluate classification models for camera trap studies (Gomez, Diez et al., 2016; Norouzzadeh et al., 2018; Swanson et al., 2015). We suggest the optimization of customized models be based more on F‐1 score rather than relying on accuracy alone, because accuracy can be heavily biased by TNs (Wolf & Jolion, 2006). This can be seen in the greater than 20% difference between our test accuracy (TNs excluded) and validation accuracy (TNs included).

The metrics used to optimize a model will depend on the purpose of the project and the resources available to the researcher. The F‐1 score can be broken down into precision and recall, both of which can be optimized for different purposes. In a study focusing on rare species (e.g., Alexander et al., 2016; Karanth, 1995), precision should be optimized to ensure the detection of all possible occurrences of animals. Alternatively, recall should be optimized if processing time is limited and every image of an animal is not essential for the global analysis. Optimizing recall is ideal for a general survey of common, easily identified animals (e.g., Chitwood et al., 2020).

### 4.3 Optimizing model performance

Analyzing model performance during training is especially useful to determine which classes the model is not identifying properly and is easily visualized using IOU graphs. Precision during training did not seem to depend on the number of images used to train each class; rather, the type of object the class refers to was most important in determining the performance of the model. Objects with unique shapes, color patterns, and textures (e.g., turkey and armadillo) were detected by the model more easily (Figure 6). The model was not as successful with objects that were small and difficult to distinguish from the background (e.g., gray squirrel), were similar to another class (e.g., coyote and dog), or when trained examples were highly variable in the subjects within the same class (e.g., humans and vehicles).

Depending on the aim of the study, the choice of metric allows the researcher to facilitate either an ID or CL model. Certain camera trap studies benefit greatly from automating the removal of TNs, especially when focusing on topics such as camera trap effectiveness (e.g., Edwards et al., 2016; Ferreira‐Rodríguez & Pombal, 2019) or instances where human‐supervised processing will be required to extract details such as behavior. To focus a model on detection of objects rather than classification, researchers should focus on metrics associated with ID. The use of this type of identification model would allow researchers to decrease processing time and ensure detection of objects while not being overly concerned with the accuracy of species classification by the model. Alternatively, studies focusing on general ecosystem monitoring (e.g., Jiménez et al., 2010; Steenweg et al., 2017) or density of common species (e.g., Parsons et al., 2017) would benefit from a CL model and should use CL metrics to build a model fully capable of both identifying and classifying species.

Several methods may be employed to adjust the model's parameters. CTs are a simple way to calibrate a model to reach the desired metric's optimal value. If optimization cannot be reached by adjustments of CTs, the model can be further improved by adding images to classes which the model consistently predicts incorrectly. Images should be added to the model's train and test directories based on performance during training (examining IOU graphs) and in the test and validation evaluation metrics. This will help the model to learn from the dataset and improve its performance on objects classification.

Establishing methods to quickly and accurately process camera trap data will allow researchers to monitor wildlife populations more autonomously. As biodiversity declines worldwide (Kolbert, 2014), employing commonly used computer science techniques in future camera trap studies will greatly enhance our ability to monitor wildlife populations.

## 5 CONCLUSIONS

1. Transfer learning with bounding boxes is successful and requires far fewer training images than traditional model building.
2. Identification and classification models built using transfer learning and small image sets can be very successful with species that are easily distinguished. However, there are cases in which species that are considered more difficult to distinguish can also be identified by using these methods.
3. The traditional metric of accuracy can give a false sense of confidence in a model because of inflation by true negatives. F‐1 should be used more commonly for general purposes because it is not biased by true negatives.
4. Studies focusing on simply removing true negatives do not require the large number of images and resources compared to studies attempting to classify species do.

## CONFLICT OF INTEREST

The authors declare no conflicts of interest.

## AUTHOR CONTRIBUTIONS

**Matthew T. Duggan:** Conceptualization (lead); Data curation (equal); Formal analysis (lead); Funding acquisition (supporting); Investigation (supporting); Methodology (lead); Project administration (equal); Resources (supporting); Software (lead); Supervision (supporting); Validation (lead); Visualization (lead); Writing‐original draft (lead); Writing‐review & editing (equal). **Melissa F. Groleau:** Conceptualization (equal); Data curation (lead); Formal analysis (equal); Funding acquisition (supporting); Investigation (equal); Methodology (supporting); Project administration (lead); Resources (supporting); Software (supporting); Supervision (lead); Validation (equal); Visualization (equal); Writing‐original draft (equal); Writing‐review & editing (equal). **Ethan P. Shealy:** Data curation (equal); Investigation (equal); Writing‐review & editing (equal). **Lillian S. Self:** Data curation (supporting); Investigation (supporting); Validation (supporting); Writing‐review & editing (supporting). **Taylor E. Utter:** Data curation (supporting); Investigation (supporting); Validation (supporting); Writing‐review & editing (supporting). **Matthew M. Waller:** Data curation (supporting); Investigation (supporting); Project administration (supporting); Supervision (supporting); Writing‐review & editing (supporting). **Bryan C. Hall:** Conceptualization (supporting); Funding acquisition (equal); Project administration (equal); Resources (equal); Writing‐review & editing (supporting). **Chris G. Stone:** Conceptualization (supporting); Funding acquisition (supporting); Investigation (supporting); Resources (supporting); Writing‐review & editing (supporting). **Layne L. Anderson:** Conceptualization (supporting); Funding acquisition (supporting); Investigation (supporting); Resources (supporting); Writing‐review & editing (supporting). **Timothy A. Mousseau:** Conceptualization (lead); Data curation (equal); Formal analysis (equal); Funding acquisition (lead); Investigation (supporting); Methodology (lead); Project administration (lead); Resources (lead); Software (supporting); Supervision (equal); Validation (supporting); Visualization (supporting); Writing‐original draft (supporting); Writing‐review & editing (equal).

## Supporting information

Appendix S1–S4

Figure S1

Figure S2

Figure S3

Figure S4

## Data Availability

The raw images used for this study are available upon request from the corresponding author (Timothy Mousseau, mousseau@sc.edu) or may be accessed directly from https://drive.google.com/drive/folders/1Dljzj4utlxSUaZ4VKFItCa0u3AibGyl_?usp=sharing.
