# Supplementary material for: An approach to rapid processing of camera trap images with minimal human input
Source: Ecol Evol. 2021 Aug 2;11(17):12051–63. doi: 10.1002/ece3.7970 (PMC8427629; doi:10.1002/ece3.7970)
Supplement: Supplementary file 1 — Appendix S1–S4 [file ECE3-11-12051-s002.docx]

# Supplementary Information

### Appendix 1: Camera Trap Study

The images used in this study were all pulled from an ongoing camera trap study on South Carolina Army National Guard (SCARNG) property. This camera trap study covers two locations in the midlands of South Carolina (SI Figs 1 and 2) with the same habitat types and species composition (SI Table 1). Images were collected and hand-processed by students at the University of South Carolina from McCrady SCARNG training center since October 2017 and from Clark’s Hill SCARNG training center since January 2019. Details for each site can be found in SI Table 2.


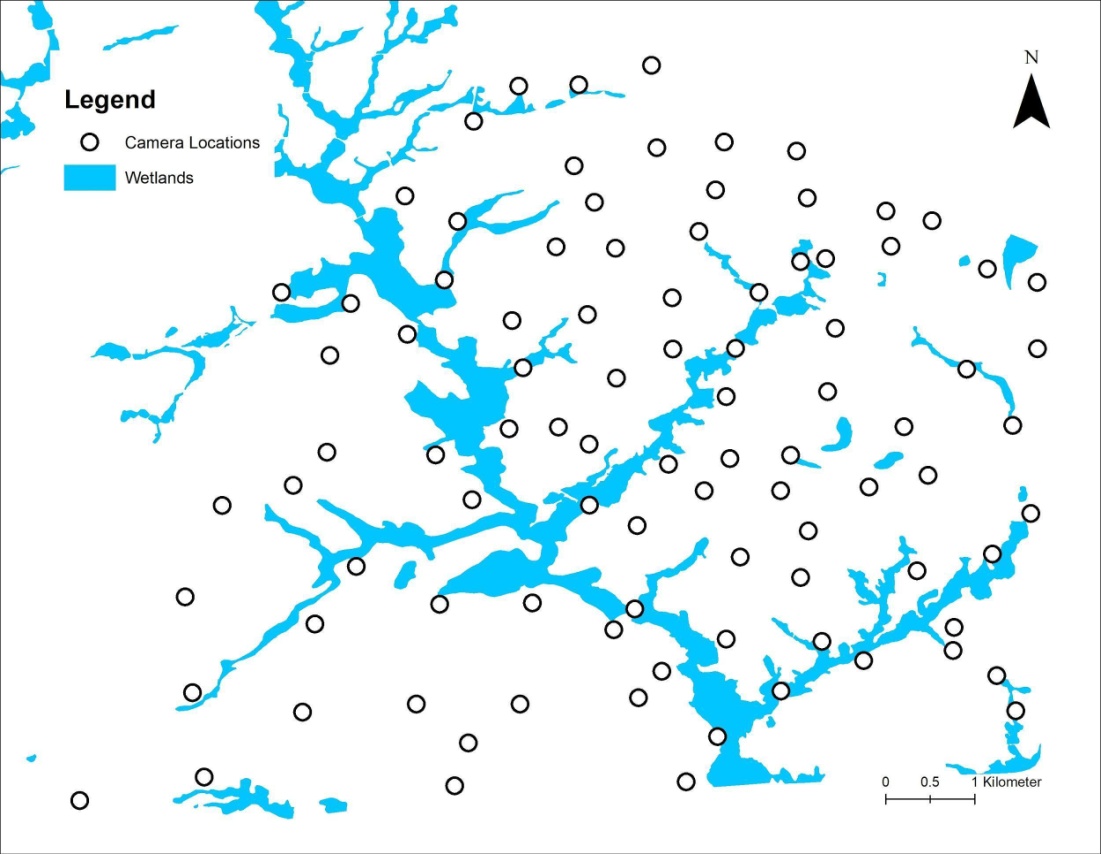


SI Figure 1. Map of camera locations at McCrady SCARNG training center in South Carolina.


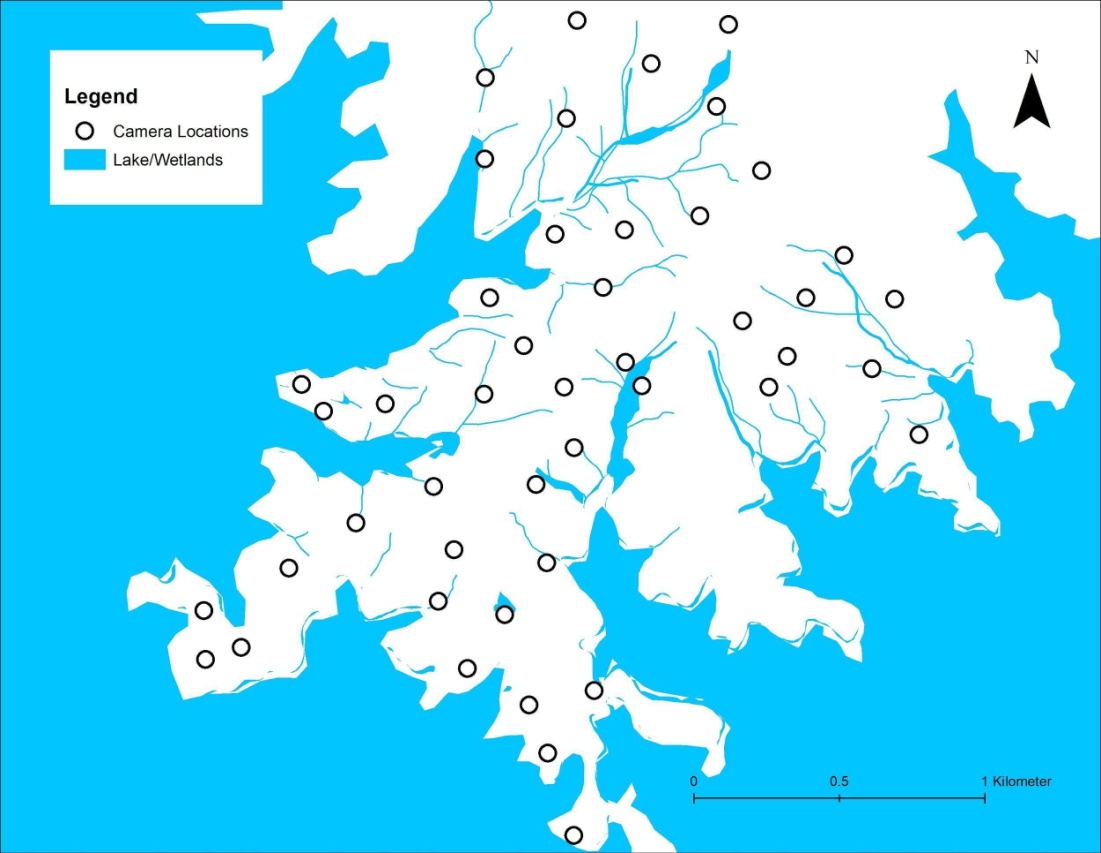


SI Figure 2. Map of camera locations at Clark’s Hill SCARNG training center in South Carolina.

SI Table 1. Reference sheet for the name of fauna species and their binomial nomenclature in South Carolina.

| **Common Name Key** | |
| --- | --- |
| Common Name | Binomial Nomenclature |
| Armadillo | *Dasypus novemcinctus* |
| Bobcat | *Lynx rufus* |
| Coyote | *Canis latrans* |
| Crow | *Corvus brachyrhynchos* |
| Deer | *Odocoileus virginianus* |
| Dog | *Canis familiaris* |
| Fox Squirrel | *Sciurus niger* |
| Grey Fox | *Urocyon cinereoargenteus* |
| Grey Squirrel | *Sciurus carolinensis* |
| Heron | *Ardea herodias* |
| Human | *Homo sapiens* |
| Opossum | *Didelphis virginiana* |
| Rabbit | *Sylvilagus floridanus* |
| Raccoon | *Procyon lotor* |
| Skunk | *Mephitis mephitis* |
| Turkey | *Meleagris gallopavo* |

SI Table 2. Study details for McCrady and Clark’s Hill SCARNG training centers in South Carolina.

|  | | **McCrady** | **Clark's Hill** |
| --- | --- | --- | --- |
| Study Began | | October 2017 | January 2019 |
| Total Species | | 24 | 24 |
| Total Individuals | | 78,359 | 4,002 |
| Total Mammal Species | | 18 | 15 |
| **Individuals of Common Species** | *Armadillo* | 205 | 113 |
|  | *Boar* | 1 | 0 |
|  | *Bobcat* | 65 | 10 |
|  | *Coyote* | 1,075 | 123 |
|  | *Deer* | 66,128 | 2,340 |
|  | *Fox Squirrel* | 1,776 | 70 |
|  | *Grey Fox* | 153 | 121 |
|  | *Grey Squirrel* | 1,127 | 510 |
|  | *Opossum* | 187 | 12 |
|  | *Rabbit* | 247 | 171 |
|  | *Raccoon* | 965 | 123 |
|  | *Turkey* | 3,352 | 123 |

### Appendix 2: Bounding Boxes

We used bounding boxes to establish ground truths in our study to increase the value of images, allowing us to use far fewer images to train our model. Bounding boxes provide the model with the location of each object dictating the bounds of the object and background noise (SI Fig. 3, human labeled). Providing the model with images without bounding boxes makes it more difficult for the model to distinguish commonality in patterns of similar objects and would further complicate identification when repeated, uncorrelated, confounding objects or background noise are present.

Once trained, the model will identify and classify all objects by placing bounding boxes: a box, the corresponding label of that object, and a feature score. A feature score is the percent likelihood that the object detected reflects the respective label. Our model correctly identified the objects in images 1-3. The model can be more precise than human labelers in finding objects, for example, image 6 displays correctly labeled tail feathers of a turkey that were not labeled correctly by human labelers. Additionally, the model may pick up objects incorrectly (image 5) with low confidence. The confidence threshold (CT) was set at 50%, so any objects detected with over 50% confidence were displayed. This CT can be adjusted to negate low confidence objects, but during training can give insights into errors that may impact validation accuracies and F-1 score. For example, in image 5, images with the same background or images of grey squirrel can be added to further distinguish the misidentified object. Image 4 shows an example of object splitting, when one object is identified by two bounding boxes. Object splitting creates problems with counting the correct number of individuals in an image. Again, adding additional similar images of an event where object splitting can occur can increase the chances of correct bounding boxes. These types of discrepancies suggest the need for a combination of human labelers and AI prescreening for a completely thorough analysis of camera trap imagery.

| 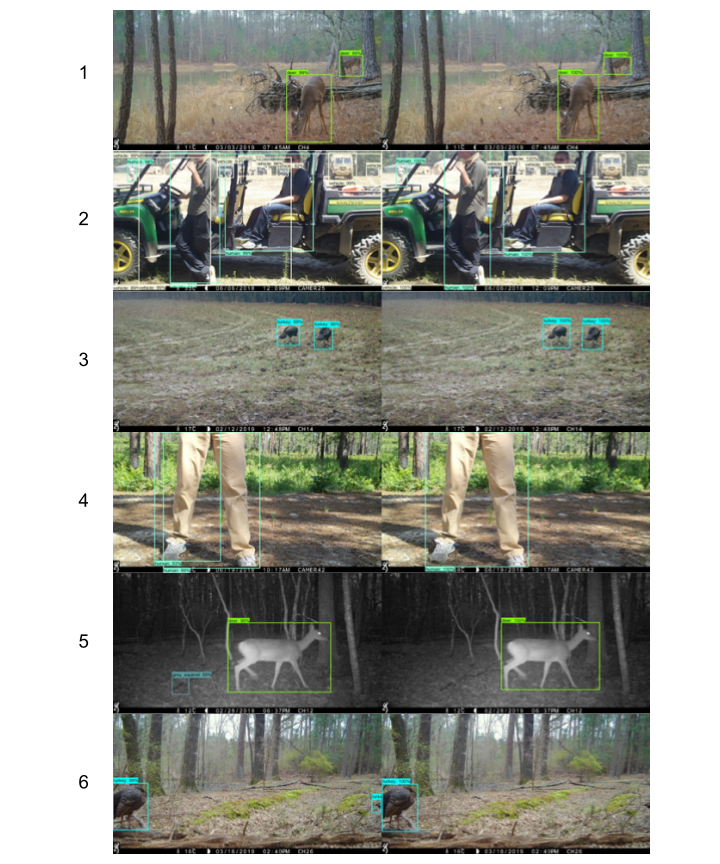 |
| --- |

SI Figure 3. Six images were randomly selected from the test set during training that evaluate the performance of training on the final step (50,000). On each image, the left side is the computer-generated image and the right side is the human labeled image.

### Appendix 3: Intersection over union

The model was evaluated throughout the training process using intersection over union (IOU), the degree of overlap between human labeled and computer-generated identifications. Higher IOU represents a greater overlap of the two. For our model, IOU did not depend on the number of images input for training; rather, the uniqueness of objects due to shape and texture was the determining factor. IOU graphs for all object classes are displayed in Supplementary material Fig. 4.


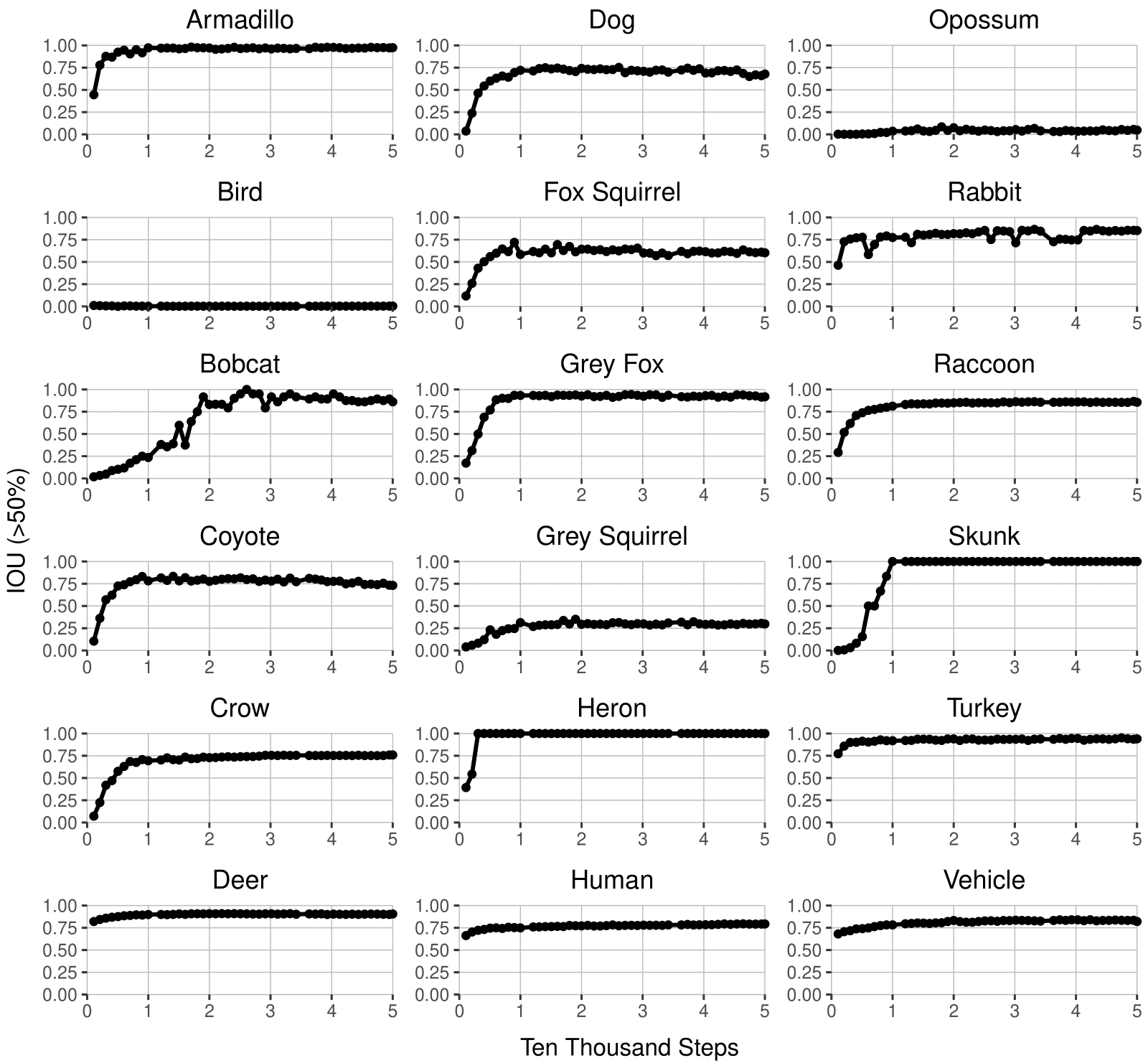


SI Figure 4. All 17 classes’ intersection over union (IOU) graphs of greater than 50% overlap over 50,000 steps conducted in training. A class for ‘Bird’ was also created but was not included in our analyses due to birds belonging to several species of songbirds and too few images.

### Appendix 4: Brief Study Comparison

When compared to methods and practices employed by other studies, our model was comparable against larger studies and performed exceedingly well across this brief survey of some of the top camera trap methodologies. These observations are summarized in SI Table 3, yet we advise the reader to heavily consider the differences between the studies and architectures as the information is examined. For future direction, a model which utilizes careful consideration of images, multi-tasking, image transformation, and ensemble learning is the best model; however, transfer learning is an efficient tool for the smaller study.

SI Table 3. Comparison of select studies that focused on camera trap fauna detection and classification. The first row is the present manuscript. Studies were chosen based on if they were published after the year 2015 and included as a reference in the present study. Our study uses approximately 74% fewer images per class then the next smallest image dataset. We demonstrate the accuracy (total objects correctly identified/total number of objects) and F1 ($2\times\frac{Precision \times Recall}{Precision + Recall}$ ) which is equal to two times the number of objects correctly identified all over two times the number of objects correctly identified plus objects not correctly identified plus detections when there were no objects.

|  | | | **Test** | | **Validation** | |  |  |  |
| --- | --- | --- | --- | --- | --- | --- | --- | --- | --- |
| **Class Type** | **Number of Training Images** | **Number of Classes** | **Accuracy** | **F1** | **Accuracy** | **F1** | **Comments** | **Dataset** | **Citation** |
| Animals & Humans | 5,277 | 17 | 71% | 83% | 92% | 76% | Limited training set and comparable F1 | McCrady and ClarksHill, SC | Duggan et al. 2021 |
| Animals | 29,176 | 26 | 89% | Not Noted | Not Noted | Not Noted | Balanced dataset, segmented images, images contained foreground animals only, and metrics are from training. | Snapshot Serengeti | Gomez et al. 2016 |
| Animals | 3.2 million | 48 | 95% | 76% (IV model) | Not Noted | Not Noted | Single-label classification, but utilizes multi-task learning. | Snapshot Serengeti | Norouzzadeh et al. 2018 |
| Animals | 3.7 million | 27 | 98% | 98% | 82% | 95% | Single-label classification, same training scripts as Norouzzadeh et al. 2018. | Undefined: U.S. & Canada | Tabak et al. 2018 |
| Animals & Humans | 47, 279 | 55 | 96.00% | 81.00% | 71.00% | 71% | Utilizes six models with low accuracy, regarding validation metrics. | Parks Canada | Schneider et al. 2020 |
