# Supplementary figures and images for: An approach to rapid processing of camera trap images with minimal human input
Source: Ecol Evol. 2021 Aug 2;11(17):12051–63. doi: 10.1002/ece3.7970 (PMC8427629; doi:10.1002/ece3.7970)

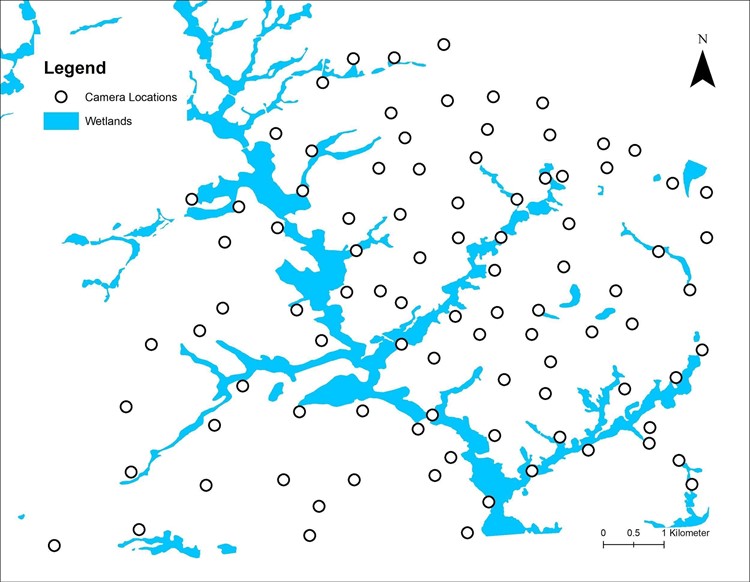

Supplement: Supplementary file 2 — Figure S1 [file ECE3-11-12051-s003.jpg]

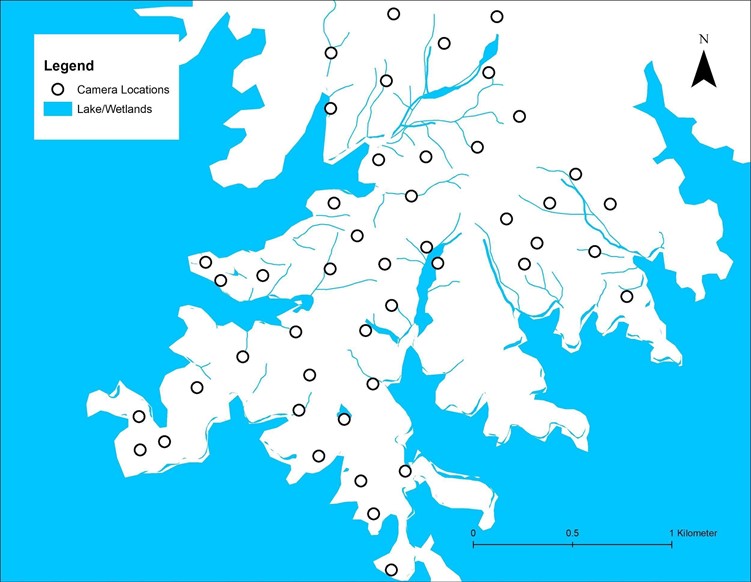

Supplement: Supplementary file 3 — Figure S2 [file ECE3-11-12051-s001.jpg]

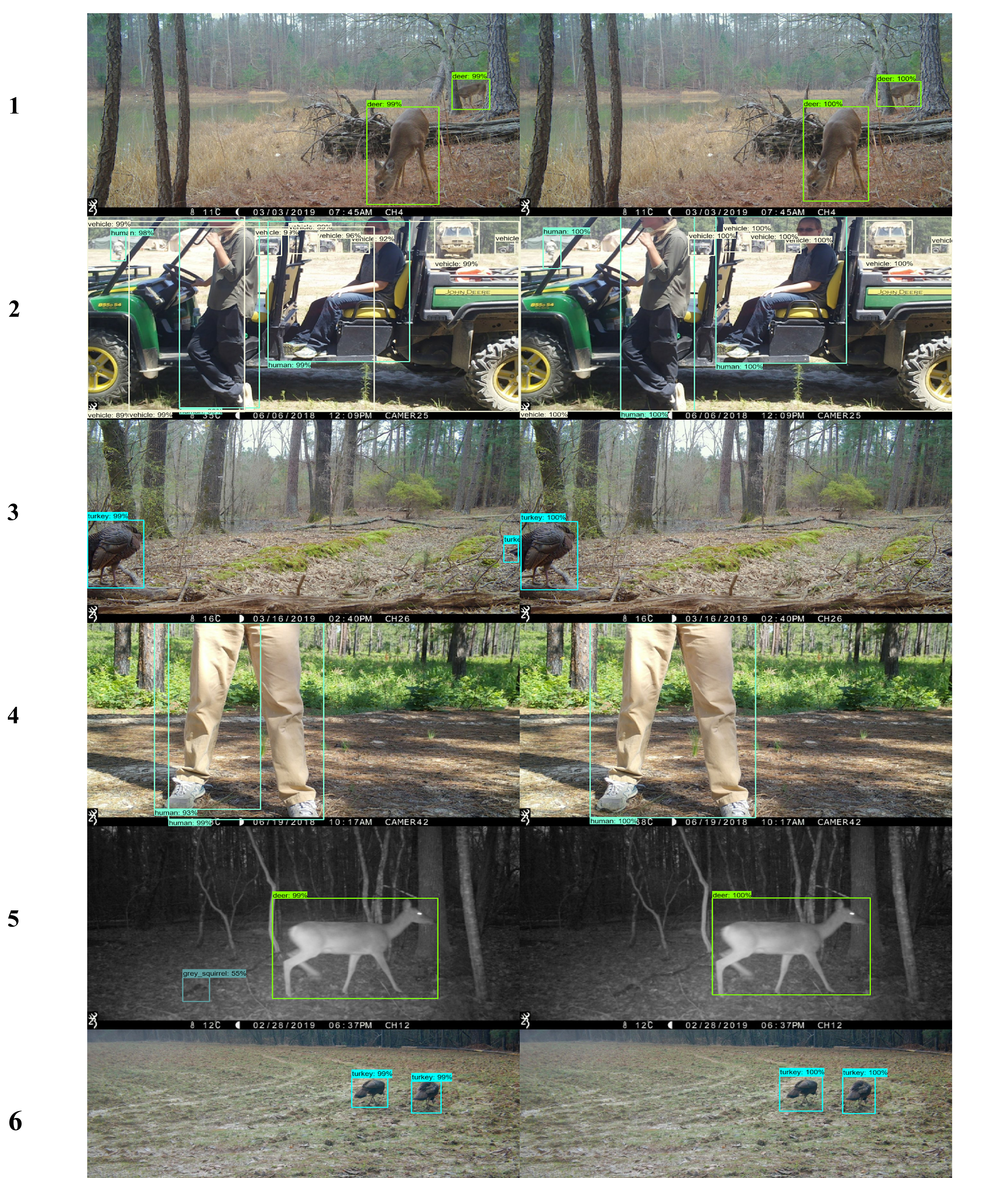

Supplement: Supplementary file 4 — Figure S3 [file ECE3-11-12051-s004.jpg]

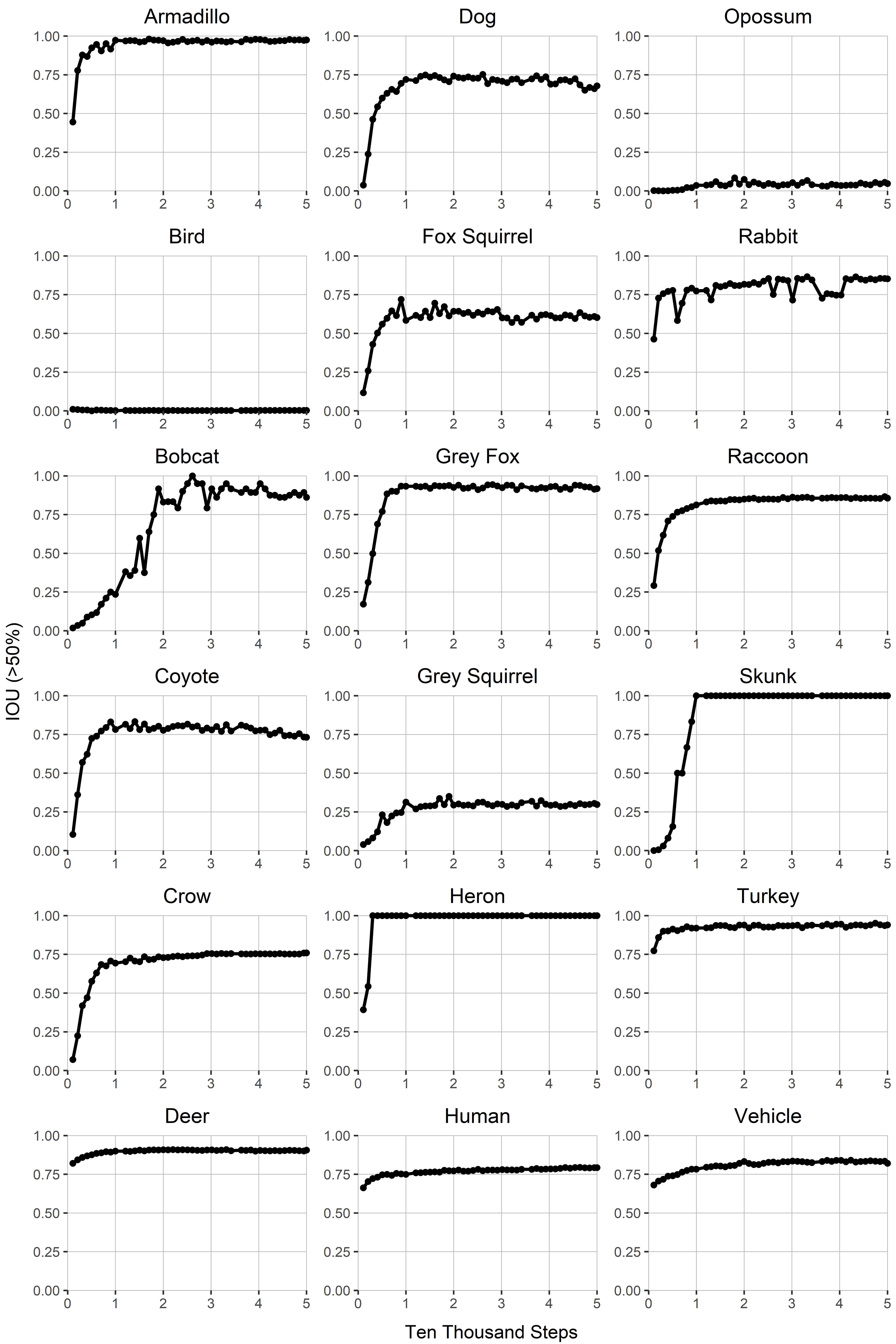

Supplement: Supplementary file 5 — Figure S4 [file ECE3-11-12051-s005.jpg]
